# Long‐Term Safety and Efficacy of Crisugabalin for Diabetic Peripheral Neuropathic Pain: A 52‐Week, Multicenter, Single‐Arm Trial

**DOI:** 10.1155/jdr/2960736

**Published:** 2026-03-23

**Authors:** Xiaohui Guo, Kailiang Wang, Tingting Zhang, Jianhua Ma, Yukun Li, Chengxia Jiang, Jie Liu, Yawei Zhang, Fang Bian, Fang Zhang, Weijuan Liu, Xiaohong Wu, Xulei Tang, Yihua Wang, Tao Ning, Shuguang Pang, Ya Li, Lijun Wang, Jia Sun, Honglin Hu, Hong Mao, Tianrong Pan, Yufeng Li, Xin Sun, Ping Li, Fangqiong Li, Qin Huang, Yaming Li

**Affiliations:** ^1^ Department of Endocrinology, Peking University First Hospital, Beijing, China, pkufh.com; ^2^ Department of Endocrinology, Emergency General Hospital, Beijing, China; ^3^ Department of Endocrinology, Nanjing First Hospital, Nanjing Medical University, Nanjing, Jiangsu Province, China, njmu.edu.cn; ^4^ Department of Endocrinology, The Third Hospital of Hebei Medical University, Shijiazhuang, Hebei Province, China, hebmu.edu.cn; ^5^ Department of Endocrinology, The Second People’s Hospital of Yibin, Yibin, Sichuan Province, China; ^6^ Department of Endocrinology, The First Affiliated Hospital of Henan University of Science and Technology, Luoyang, Henan Province, China, haust.edu.cn; ^7^ Department of Endocrinology, Pingxiang People’s Hospital, Pingxiang, Jiangxi Province, China, pxsrmyy.cn; ^8^ Department of Endocrinology, Cangzhou People’s Hospital, Cangzhou, Hebei Province, China, czrmyy.com; ^9^ Department of Endocrinology, Kaifeng Hospital of Traditional Chinese Medicine, Kaifeng, Henan Province, China, kfszyy.cn; ^10^ Department of Endocrinology, Chongqing University Three Gorges Hospital, Chongqing, China, cqu.edu.cn; ^11^ Department of Endocrinology, Zhejiang Provincial People’s Hospital, Hangzhou, Zhejiang Province, China, hospitalstar.com; ^12^ Department of Endocrinology, The First Hospital of Lanzhou University, Lanzhou, Gansu Province, China, lzu.edu.cn; ^13^ Department of Endocrinology, Nanyang First People’s Hospital, Nanyang, Henan Province, China; ^14^ Department of Endocrinology, Baotou Central Hospital, Baotou, Inner Mongolia Autonomous Region, China, btzxyy.com; ^15^ Department of Endocrinology, Jinan Central Hospital Affiliated to Shandong University, Jinan, Shandong Province, China, sdu.edu.cn; ^16^ Department of Endocrinology, First Affiliated Hospital of Xi’an Medical College, Xi’an, Shaanxi Province, China; ^17^ Department of Endocrinology, Taizhou First People’s Hospital, Taizhou, Zhejiang Province, China; ^18^ Department of Endocrinology, Zhujiang Hospital of Southern Medical University, Guangzhou, Guangdong Province, China, zjyy.com.cn; ^19^ Department of Endocrinology, The First Affiliated Hospital of Anhui Medical University, Hefei, Anhui Province, China, ahmu.edu.cn; ^20^ Department of Endocrinology, The Central Hospital of Wuhan, Wuhan, Hubei Province, China, zxhospital.com; ^21^ Department of Endocrinology, The Second Affiliated Hospital of Anhui Medical University, Hefei, Anhui Province, China, ahmu.edu.cn; ^22^ Department of Endocrinology, Beijing Pinggu District Hospital, Beijing, China; ^23^ Department of Neurology, The First Hospital of Jilin University, Changchun, Jilin Province, China, jlu.edu.cn; ^24^ Department of Endocrinology, Yuncheng Central Hospital, Yuncheng, Shanxi Province, China, ycch.cn; ^25^ Medical Department, Haisco Pharmaceutical Group Co., Ltd., Chengdu, Sichuan Province, China

## Abstract

**Introduction:**

The HSK16149‐201/301 trial demonstrated short‐term efficacy of the GABA analog crisugabalin for diabetic neuropathic pain.

**Methods:**

In the current study, patients in both the crisugabalin and placebo control groups of the HSK16149‐201/301 trial were invited to receive 80 mg/day crisugabalin for an additional 52 weeks. The primary end point was safety. The secondary end point was pain control as measured using the Short‐Form McGill Pain Questionnaire (SF‐MPQ). A total of 301 patients (mean age 59.7 years and males 58.1%) were enrolled.

**Results:**

The rate of treatment‐related adverse events (TRAEs) was 38.9% (117/301). The most frequent TRAEs were dizziness (27.2%), somnolence (8.3%), and peripheral edema (3.0%). The rate of Grade 3 or higher TRAEs was 1.7%. TRAEs led to dose reduction in 41 patients (13.6%), treatment interruption and discontinuation each in three patients (1.0%). At week 52, the mean difference in SF‐MPQ pain rating index (PRI) and visual analog scale pain score from baseline was −2.5 (95% CI −2.9 to −2.0; paired *t*‐test *p* < 0.0001) and −23.4 (95% CI −25.6 to −21.2; paired *t*‐test *p* < 0.0001), respectively. The proportion of patients with SF‐MPQ PPI score ≤ 1 increased by 19.0% over baseline (*p* < 0.0001).

**Conclusions:**

In summary, crisugabalin at 80 mg/day was well tolerated and demonstrated sustained analgesic activities.

**Trial Registration:**

ClinicalTrials.gov identifier: NCT05890053

## 1. Introduction

Diabetes is increasingly common with rapid aging of the population and changes in lifestyle [1, 2]. In line with this trend, diabetic peripheral neuropathy now ranks fifth in age‐standardized disability‐adjusted life years (DALYs) among all neurological conditions [3]. Approximately 30% of patients with diabetic peripheral neuropathy will eventually experience moderate to severe unremitting neuropathic pain [4]. Nortriptyline, duloxetine, pregabalin, and gabapentin are recommended as first‐line treatments in patients with diabetic peripheral neuropathic pain by most international guidelines [5, 6]. Pain control is inadequate in a significant proportion of patients despite the currently available treatments [7].

The calcium channel alpha‐2‐delta (*α*2*δ*) subunit has long been implicated in neuropathic pain and serves as the primary therapeutic target of gabapentinoids, including pregabalin [8], mirogabalin, and gabapentin [9]. Long‐term safety and efficacy studies showed that gabapentinoids could provide sustained improvement in neuropathic pain [10, 11]. Crisugabalin (HSK16149) is an oral GABA analog that binds to the calcium channel *α*2*δ* subunit. It exerts pain relief by inhibiting Ca^2+^ influx and the subsequent release of excitatory neurotransmitters [12–14]. Though pregabalin, mirogabalin, and crisugabalin, as *α*2*δ* ligands, share a common mechanism of modulating voltage‐gated calcium channels to alleviate neuropathic pain, they substantially differ in molecular architecture that determines their differential binding kinetics, selectivity, and pharmacological action.

Pregabalin has a flexible alkyl backbone with a carboxylic acid and primary amine, conferring moderate affinity for both *α*2*δ*‐1 and *α*2*δ*‐2 subunits [15]. The limited selectivity of pregabalin partially contributes to its prominent off‐target central nervous system (CNS) side effects such as dizziness and somnolence. Meanwhile, mirogabalin has a bicycloheptene scaffold that enhances its *α*2*δ*‐1 selectivity, contributing to its less prominent CNS side effects compared to pregabalin [16]. In contrast, crisugabalin has a distinct rigid tricyclic cage‐like structure and a phenylsulfonic acid moiety, which increases its molecular rigidity and binding to *α*2*δ*‐1 [17]. In preclinical studies, crisugabalin was 23 times more potent than pregabalin in binding to the *α*2*δ* subunit, with an IC50 of 3.96 nM versus 92.12 nM, and had more durable activity (24 h vs. 12 h) and lower CNS exposure [12].

Our in vitro data also showed that crisugabalin and mirogabalin exhibited distinct dissociation kinetics from the *α*2*δ* subunits of voltage‐gated calcium channels. Crisugabalin had a more prolonged dissociation half‐life (*t*_1/2_) from *α*2*δ*1 (77.25 min, *K*_off_ 0.009 min^−1^) than from *α*2*δ*2 (4.00 min, 0.1732 min^−1^), with a *K*_off_ ratio of 19.30. The *K*_off_ ratio was 4.85 with mirogabalin [18]. While both crisugabalin and mirogabalin favor *α*2*δ*1 over *α*2*δ*2, the tighter and more sustained engagement of crisugabalin suggests enhanced analgesic potency and reduced off‐target effects for crisugabalin compared to mirogabalin.

Crisugabalin was well tolerated in healthy subjects [19]. In a Phase 2/3 trial, crisugabalin at a daily dose of 40 or 80 mg resulted in a significant reduction in average daily pain score (ADPS), a gold standard patient‐reported outcome measure for pain [20], compared with placebo at Week 13 in patients with diabetic peripheral neuropathic pain [21]. Adverse events (AEs) with crisugabalin were mostly mild/moderate and require no therapeutic intervention. In a Phase 3 trial of postherpetic neuralgia, crisugabalin showed sustained pain‐reducing activities over 26 weeks and a good safety profile [22]. However, no long‐term data are available on the safety and efficacy of crisugabalin in patients with diabetic peripheral neuropathic pain.

Here, we report the long‐term safety and efficacy of 80 mg/day crisugabalin in a 52‐week extension period in patients who completed the 13‐week Phase 2/3 HSK16149‐201/301 trial.

## 2. Materials and Methods

The trial protocol and all amendments were approved by the Independent Ethics Committee/Institutional Review Board of each participating center (master protocol approval #2021 Drug Registration 140‐amendment, by the Ethics Committee of Biomedical Research, Peking University First Hospital). The trial was undertaken in adherence with the principles of the Declaration of Helsinki and Good Clinical Practice Guidelines. Written informed consent was obtained from all patients before any trial‐related activities. This manuscript follows the Transparent Reporting of Evaluations With Non‐randomized Designs (TREND) reporting guideline for nonrandomized studies. The formal trial protocol is provided in Protocol.

### 2.1. Study Design and Patients

Adult patients who had completed double‐blind treatment and safety follow‐up in the HSK16149‐201/301 trial, a randomized, double‐blind, placebo, and active‐controlled Phase 2/3 adaptive trial [21], were invited to enter this 52‐week, single‐arm extension trial (Figure 1a). Key exclusion criteria included poor compliance (< 80%), significant safety concerns, and AEs during the double‐blind treatment period in the HSK16149‐201/301 trial, estimated glomerular filtration rate (eGFR) < 60 mL/min/1.73 m^2^ at the end of the double‐blind treatment period, and positive hepatitis B surface antigen (HBsAg) or hepatitis C virus (HCV) antibody, human immunodeficiency virus antibody, or serum antibody to *Treponema pallidum*. The eligibility criteria are fully described in the study protocol.

Figure 1(a) Trial design. (b) Patient flow through the trial.

**Figure:**
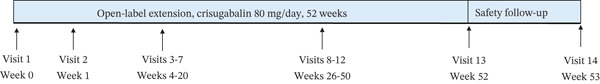
(a)

**Figure:**
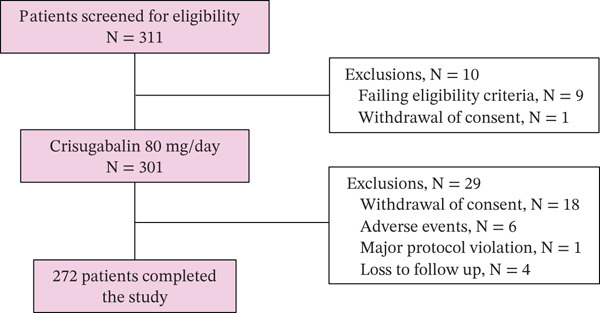
(b)

### 2.2. Treatment

Eligible patients received crisugabalin 80 mg (40 mg twice daily [BID]) for 52 weeks. The dose was based on the results of the Phase 2/3 HSK16149‐201/301 trial [21]. Dose reduction (to 20 mg BID) was allowed at the discretion of the investigators to optimize tolerability. Rescue pain relief with acetaminophen was allowed at 2.0 g/day for no more than 5 days at the discretion of investigators. No other analgesics were allowed.

### 2.3. Assessments

Blood routines, blood biochemistries, urine routine tests, and vital signs were measured at each visit. Physical examination and hemoglobin A1C measurement were conducted at baseline (Visit 1, Week 0), every 4 weeks for Visits 4–7, every 6 weeks for Visits 8–12, and upon the trial completion (Visit 13, Week 52). In addition, a 12‐lead electrocardiogram (ECG) was obtained at Visits 1 and 13. Safety was monitored throughout the study and up to 1 week after the final dose (Visit 14, Week 53). Assessments included the frequency and severity of treatment‐emergent AEs (TEAEs) and treatment‐related AEs (TRAEs). AEs were graded according to the National Cancer Institute Common Terminology Criteria for Adverse Events (NCI CTCAE) Version 5.0 and described both in terms of the current MedDRA system organ class, preferred term, and CTCAE grade and coded using MedDRA Version 26.0.

Information on concomitant medications and therapies was elicited at each visit from Week 0 to 52 and 1 week after the final dose. Drugs were classified based on the WHODrug medicinal information dictionary [23].

At each visit, patients provided a self‐assessment of pain using the Short‐Form McGill Pain Questionnaire (SF‐MPQ) [21]. The questionnaire includes the pain rating index (SF‐MPQ PRI), which consists of 3 subscales (sensory score [11 items], affective score [4 items], and total scores [15 items]) and is rated on an intensity scale (0 [*none*] to 3 [*severe*]), a 100‐mm visual analog scale (VAS), where 0 corresponds to *no pain* and 100 corresponds to *worst possible pain*, and present pain intensity (SF‐MPQ PPI) on a scale of 0 (*no pain*) to 5 (*worst pain*) [24].

### 2.4. Statistical Analysis

A study population of 300 patients was planned for the trial. Safety measures were analyzed in a modified intention‐to‐treat (ITT) population that included all patients who had received at least one dose of crisugabalin. Efficacy measures were analyzed in patients with at least one postbaseline efficacy evaluation. Missing efficacy data at Week 52 were imputed using the last observation carried forward (LOCF) method. The primary end point was safety, and the secondary end point was the efficacy measure as described using SF‐MPQ.

Statistical analysis was undertaken using SAS EG 8.3 (The SAS Institute, Cary, NC).

## 3. Results and Discussion

### 3.1. Patient Characteristics

Flow of the patients through the trial is shown in Figure 1b. Of the 729 patients enrolled in the HSK16149‐201/301 trial, 644 had completed double‐blind treatment and safety follow‐up, and between February 17, 2022, and July 19, 2023, among 311 patients who were screened for eligibility for the current extension trial, 301 were enrolled, and 272 (90.4%) completed the trial as planned. The mean age of the enrolled patients was 59.7 ± 9.0 years, and 58.1% were male. The median duration of diabetic peripheral neuropathic pain was 27.7 months (Q1, Q3 18.3, 43.0). Patient characteristics are detailed in Table 1.

**Table 1:** Demographic and baseline characteristics of the patients.

Characteristics	*N* = 301
Mean (SD) age, years	59.7 (9.0)
Sex	
Male	175 (58.1)
Female	126 (41.9)
Mean (SD) height (cm)	164.9 (8.1)
Mean (SD) body weight (kg)	67.3 (11.4)
Mean (SD) body mass index (kg/m^2^)	24.7 (3.1)
Type 2 diabetes	279 (92.7)
Median (Q1,Q3) duration of diabetes neuropathic pain (months)	27.7 (18.3, 43.0)
HbA1c (%), mean (SD)	7.4 (1.3)
Mean (SD) duration of DPNP (months)	39.3 (34.46)
SF‐MPQ^a^	
VAS, mean (SD)	45.3 (15.2)
PRI, mean (SD)	5.6 (4.6)
PPI	
≤ 1	206 (68.4)
2	68 (22.6)
≥ 3	27 (9.0)

*Note:* Data are expressed as *N* (%) unless otherwise specified.

^a^Short‐Form McGill Pain Questionnaire (SF‐MPQ) includes the pain rating index (PRI), which consists of 3 subscales (sensory score [11 items], affective score [4 items], and total scores [15 items]) and is rated on an intensity scale (0 [*none*] to 3 [*severe*]), a 100‐mm visual analog scale (VAS), where 0 corresponds to *no pain* and 100 corresponds to *worst possible pain*, and present pain intensity (PPI) on a scale of 0 (*no pain*) to 5 (*worst pain*).

Treatment characteristics are described in Table S1. The mean duration of exposure to crisugabalin was 340.1 ± 71.8 days, with a mean relative dose intensity of 99.8*%* ± 2.9*%*. Most patients (98.3%) had a compliance rate between 80% and 120%. Forty‐seven patients (15.6%) had dose reduction, and 127 (42.2%) experienced dose interruption.

### 3.2. Safety

The rate of TEAEs was 86.4% (260/301). The most frequent TEAEs were dizziness (29.2%), COVID‐19 (23.3%), and body weight increased (12.6%). Dose reduction due to TEAEs occurred in 43 patients (14.3%). TEAEs led to dose interruption in nine patients (3.0%) and treatment discontinuation in seven patients (2.3%) (Table S2). Six patients (2.0%) withdrew due to TEAEs. One patient died of a traffic accident during the trial period.

The rate of TRAEs was 38.9% (117/301). The most frequent TRAEs were dizziness (27.2%), somnolence (8.3%), and peripheral edema (3.0%). Grade 3 or higher TRAEs were infrequent (1.7%) and included dizziness (1.0%), peripheral edema (0.3%), and gastroesophageal reflux disease (0.3%) (Table 2). TRAEs led to dose reduction in 41 patients (13.6%) and dose interruption and treatment discontinuation each in 3 patients (1.0%). Two patients (0.7%) withdrew due to TRAEs. No treatment‐related death was reported.

**Table 2:** Treatment‐related adverse events (TRAEs) in the modified intent‐to‐treat population.

	Any grade	≥ Grade 3
TRAEs	117 (38.9)	5 (1.7)
TRAEs leading to dose reductions	41 (13.6)	
TRAEs leading to dose interruptions	3 (1.0)	
TRAEs leading to treatment discontinuations	3 (1.0)	
TRAEs leading to study terminations	2 (0.7)	
TRAEs leading to death	0	
Serious TRAEs	5 (1.7)	
TRAEs		
Dizziness	82 (27.2)	3 (1.0)
Somnolence	25 (8.3)	—
Peripheral edema	9 (3.0)	1 (0.3)
Body weight increased	8 (2.7)	—
Fatigue	6 (2.0)	—
Nausea	5 (1.7)	—
Vomiting	4 (1.3)	—
Hepatic function abnormalities	4 (1.3)	—
Bile acid increased	3 (1.0)	—
Hyperlipidemia	3 (1.0)	—
Abnormal gait	3 (1.0)	—
Gastroesophageal reflux disease	1 (0.3)	1 (0.3)
Serious TRAEs		
Dizziness	3 (1.0)	
Gastroesophageal reflux disease	1 (0.3)	
Peripheral edema	1 (0.3)	

### 3.3. Efficacy Measure

The SF‐MPQ PRI started to decline from 1 week posttreatment with crisugabalin (Figure 2a). At Week 52, the mean difference from baseline was −2.5 (95% CI −2.9 to −2.0; paired *t*‐test *p* < 0.0001). The proportion of patients with an SF‐MPQ PPI ≥ 3 was 9.0% and 3.0% at baseline and Week 52, respectively; the proportion of patients with an SF‐MPQ PPI ≤ 1 was 68.4% and 87.4% at baseline and Week 52, respectively (Wilcoxon test *p* < 0.0001 for both) (Figure 2b). The reduction in SF‐MPQ VAS at Week 52 from baseline was −23.4 (95% CI −25.6 to −21.2, paired *t*‐test *p* < 0.0001) (Figure 2c).

Figure 2Efficacy of crisugabalin 80 mg/day over time for diabetic peripheral neuropathic pain. Mean changes from baseline in Short‐Form McGill Pain Questionnaire (SF‐MPQ) pain rating index (PRI) in (a) and visual analog scale (VAS) scores in (c) are shown as the time course of mean changes with standard error. The proportions of patients with SF‐MPQ present pain intensity (PPI) scores ≤ 1, 2, and ≥ 3 over time are shown in (b). Data were analyzed in patients with at least one posttreatment efficacy evaluation. Missing efficacy data at Week 52 were imputed using the last observation carried forward method.

**Figure:**
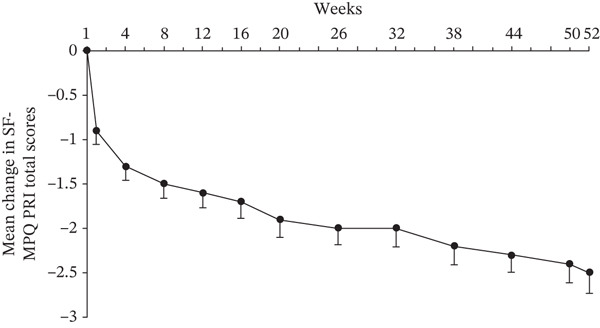
(a)

**Figure:**
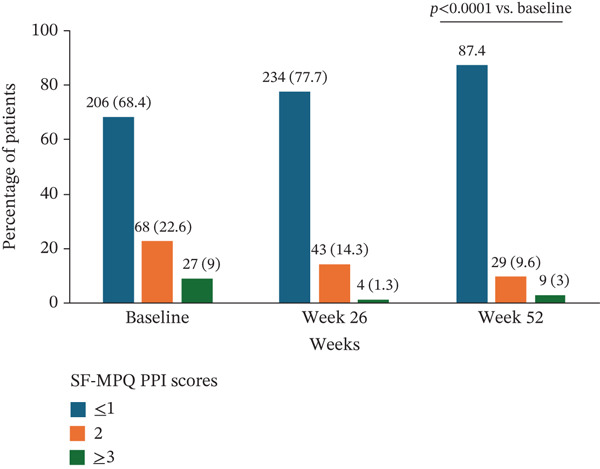
(b)

**Figure:**
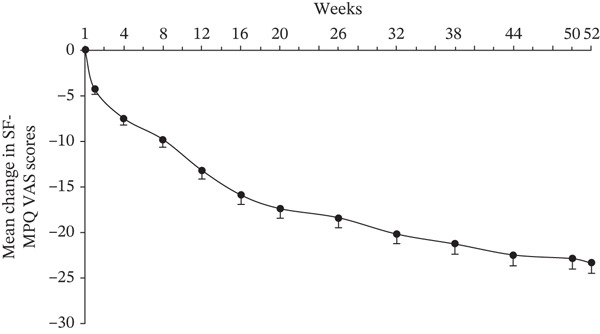
(c)

In this 52‐week, open‐label, extension trial, crisugabalin at a dose of 80 mg/day demonstrated an overall acceptable safety profile, with no unexpected AEs. Grade 3 or higher dizziness occurred in 1.0% of the patients, and abated after treatment was discontinued. Grade 3 or higher somnolence was not reported. Crisugabalin led to a significant reduction in all three measures of SF‐MPQ at Week 52, indicating sustained pain relief. These findings encourage the long‐term use of crisugabalin for diabetic peripheral neuropathic pain.

Approximately half of patients with diabetes have peripheral neuropathy, and roughly 30% of these patients will eventually develop neuropathic pain [25]. These patients often require long‐term treatment; accordingly, the safety of the pharmacotherapy is of paramount importance. A meta‐analysis of randomized controlled trials showed that pregabalin could alleviate neuropathic pain but was associated with a variety of AEs that may lead to dose reduction or interruption, including somnolence, edema, visual disturbances, ataxia, vertigo, and euphoria [26]. In this trial, 2.3% of the patients discontinued treatment due to TEAEs compared to an overall discontinuation rate of 20% with pregabalin [26]. The rate of treatment discontinuation due to TRAEs in this trial was also lower than with pregabalin at 600 mg/day (1.0% vs. 16.0%) [27]. In a study based on pooled data by Ogawa et al., the rate of treatment discontinuation with pregabalin treatment due to dizziness and somnolence was 23.5% and 10.9%, respectively, in trials in Japan, and 16.0% and 34.2%, respectively, in trials in Western countries [28]. In contrast, the rate of treatment discontinuation due to dizziness in this trial was only 1.0%; no patients discontinued treatment due to somnolence. Serious AEs and TRAEs were infrequent and required no interventions in the majority of the cases in this trial. This trial adds important evidence with regard to the long‐term safety of crisugabalin, substantiating the overall good safety profile of crisugabalin.

In the HSK16149‐201/301 trial (NCT04647773), crisugabalin at 80 mg/day led to a significantly greater mean change in ADPS at Week 13 from baseline compared to placebo (−2.16 vs. −1.23, *p* < 0.001) in patients with diabetic peripheral neuropathic pain [21]. At Week 52 in this trial, the mean difference from baseline was −2.5 and −23.4 in SF‐MPQ PRI and SF‐MPQ VAS, respectively. In contrast, the mean difference from baseline to 52 weeks of treatment with mirogabalin was −1.5 and −9.8 in the same two measures [11]. The distinct structural feature of crisugabalin partially contributes to its high target selectivity, rapid onset of analgesia, and a reduced CNS effect profile. The findings of this trial further suggest that crisugabalin could emerge as a promising candidate for next‐generation neuropathic pain therapy.

This trial has several limitations. First, the sample size (*n* = 301) is not adequate for the detection of rare AEs. Second, only Chinese patients were enrolled. Whether the findings could be extrapolated to other population requires further studies. Third, we did not assess the effects of crisugabalin on sleep disturbance and quality of life.

## 4. Conclusions

In conclusion, crisugabalin 80 mg/day (40 mg BID) was well tolerated, with no new safety concerns. Crisugabalin demonstrated sustained efficacy over the 52 weeks of treatment. The study findings support the long‐term use of crisugabalin for patients with diabetic peripheral neuropathic pain.

## Author Contributions

Conceptualization: Xiaohui Guo, Jianhua Ma, Yukun Li, and Xiaohong Wu. Data curation: Xiaohui Guo, Kailiang Wang, Tingting Zhang, Jianhua Ma, Yukun Li, Chengxia Jiang, Jie Liu, Yawei Zhang, Fang Bian, Fang Zhang, Weijuan Liu, Xiaohong Wu, Xulei Tang, Yihua Wang, Tao Ning, Shuguang Pang, Ya Li, Lijun Wang, Jia Sun, Honglin Hu, Hong Mao, Tianrong Pan, Yufeng Li, Xin Sun, and Ping Li. Formal analysis: Xiaohui Guo, Kailiang Wang, Ya Li, Chengxia Jiang, and Jie Liu. Funding acquisition: Fangqiong Li and Xiaohui Guo. Investigation: Xiaohui Guo, Jianhua Ma, Yukun Li, and Xiaohong Wu. Project administration: Xiaohui Guo, Kailiang Wang, Yawei Zhang, Fang Bian, and Weijuan Liu. Supervision: Kailiang Wang, Fangqiong Li, Qin Huang, Tianrong Pan, and Jianhua Ma. Roles/writing—original draft: Xiaohui Guo and Kailiang Wang. Writing—review and editing: Xiaohui Guo, Kailiang Wang, Tingting Zhang, Jianhua Ma, Yukun Li, Chengxia Jiang, Jie Liu, Yawei Zhang, Fang Bian, Fang Zhang, Weijuan Liu, Xiaohong Wu, Xulei Tang, Yihua Wang, Tao Ning, Shuguang Pang, Ya Li, Lijun Wang, Jia Sun, Honglin Hu, Hong Mao, Tianrong Pan, Yufeng Li, Xin Sun, Ping Li, Fangqiong Li, Qin Huang, and Yaming Li.

## Funding

The study was funded by Haisco Pharmaceutical Group Co., Ltd

## Conflicts of Interest

This trial was sponsored by Haisco Pharmaceutical Group Co., Ltd. Fangqiong Li, Qin Huang, and Yaming Li are full‐time employees of Haisco Pharmaceutical Group Co., Ltd. All other authors have declared no conflicts of interest.

## Supporting information


**Supporting information** Additional supporting information can be found online in the Supporting Information section. Table S1: Treatment characteristics. Table S2: Treatment‐emergent adverse events (TEAEs) in the modified intent‐to‐treat population.

## Data Availability

The datasets generated during and/or analyzed during the current study are available from the corresponding authors on request.
