# Supplementary material for: Long‐Term Safety and Efficacy of Crisugabalin for Diabetic Peripheral Neuropathic Pain: A 52‐Week, Multicenter, Single‐Arm Trial
Source: J Diabetes Res. 2026 Mar 23;2026:2960736. doi: 10.1155/jdr/2960736 (PMC13140441; doi:10.1155/jdr/2960736)
Supplement: Supplementary file 1 — Supporting information Additional supporting information can be found online in the Supporting Information section. Table S1: Treatment characteristics. Table S2: Treatment‐emergent adverse events (TEAEs) in the modified intent‐to‐treat population. [file JDR-2026-2960736-s001.docx]

| Supplementary Table 1. Treatment characteristics | |
| --- | --- |
| Duration of exposure, days |  |
| Mean (SD) | 340.1(71.8) |
| Median (Q1, Q3) | 362.0(360.0, 364.0) |
| Relative dose intensity (%)* |  |
| Mean (SD) | 99.8(2.9) |
| Median (Q1, Q3) | 100.0(99.9, 100.0) |
| Compliance † |  |
| <80% | 5(1.7) |
| 80%-120% | 296(98.3) |
| >120% | 0 |
| Dose modification |  |
| Yes | 47(15.6) |
| No | 254(84.4) |
| Dose interruptions |  |
| Yes | 127(42.2) |
| No | 174(57.8) |
| Dose termination |  |
| Yes | 29(9.6) |
| No | 272(90.4) |
| *Relative drug intensity (RDI) is defined as the actual dose received divided by the preset dose in the FAS.  †Compliance (%) is defined as the percentage of the prescribed doses of the medication actually taken by the patient during the study and calculated as the actual doses of a drug during the study divided by the planned doses of the drug multiplied by 100%. | |

| Supplementary Table 2. Treatment-emergent adverse events (TEAEs) in the modified intent-to-treat population | | |
| --- | --- | --- |
|  | Any grade | ≥Grade 3 |
| TEAEs | 260(86.4) | 58 (19.3) |
| TEAEs leading to dose reductions | 43(14.3) | |
| TEAEs leading to dose interruptions | 9 (3.0) | |
| TEAEs leading to treatment discontinuations | 7 (2.3) | |
| TEAEs leading to study terminations | 6 (2.0) | |
| TEAEs leading to death | 1 (0.3) | |
| Serious TEAEs | 54 (17.9) | |
| TEAEs (≥5%) |  |  |
| Dizziness | 88(29.2) | 3(1.0) |
| COVID-19 | 70(23.3) | 3(1.0) |
| Body weight increased | 38(12.6) | - |
| Upper respiratory tract infection | 33(11.0) | - |
| Hyperlipidemia | 30(10.0) | - |
| Urinary tract infection | 29(9.6) | - |
| Somnolence | 26(8.6) | - |
| Poorly controlled diabetes | 22(7.3) | 6( 2.0) |
| Fever | 22(7.3) | - |
| Hepatic function abnormalities | 19(6.3) | - |
| Hyperuricemia | 17(5.6) | - |
| Body weight decreased | 16(5.3) | - |
| Serious TEAEs (≥1%) |  |  |
| Cerebral infarction | 6(2.0) | |
| Poorly controlled diabetes | 4(1.3) | |
| Coronary arteriosclerosis | 4(1.3) | |
| Dizziness | 3(1.0) | |
| Infectious pneumonia | 3(1.0) | |
| Vitreous hemorrhage | 3(1.0) | |
| TEAEs leading to study terminations |  | |
| Dizziness | 3(1.0) | |
| Embolic infarction | 1(0.3) | |
| Infectious pneumonia | 1(0.3) | |
| Schizophrenia | 1(0.3) | |
| Data are expressed in number (%).  AEs were coded by the Medical Dictionary for Regulatory Activities (MedDRA) version 24.0 or later.  Any-grade treatment-emergent adverse events (TEAEs) occurring in over 5% of patients and ≥grade 3 TEAEs in at least 2 patients are listed. Serious TEAEs occurring in ≥1% of the patients are described. | | |
